# Fe-Cr-Nb-B Magnetic Particles and Adipose-Derived Mesenchymal Cells Trigger Cancer Cell Apoptosis by Magneto-Mechanical Actuation

**DOI:** 10.3390/nano13222941

**Published:** 2023-11-14

**Authors:** Horia Chiriac, Anca Emanuela Minuti, Cristina Stavila, Dumitru-Daniel Herea, Luminita Labusca, Gabriel Ababei, George Stoian, Nicoleta Lupu

**Affiliations:** 1 National Institute of Research and Development for Technical Physics, 700050 Iasi, Romania; cstavila@phys-iasi.ro (C.S.); dherea@phys-iasi.ro (D.-D.H.); llabusca@phys-iasi.ro (L.L.); gababei@phys-iasi.ro (G.A.); gstoian@phys-iasi.ro (G.S.); nicole@phys-iasi.ro (N.L.); 2 Faculty of Physics, “Alexandru Ioan Cuza” University, 700506 Iasi, Romania

**Keywords:** magnetic particles, magneto-mechanical actuation, cancer cells, adipose-derived mesenchymal cells, cell carriers

## Abstract

Magnetic nanoparticles (MPs) are emerging as powerful and versatile tools for biotechnology, including cancer research and theranostic applications. Stem cell-mediated magnetic particle delivery has been previously recognized as a modality to target sites of malignancies. Here, we propose the use of adipose-derived mesenchymal cells (ADSC) for the targeted delivery of Fe-Cr-Nb-B magnetic particles to human osteosarcoma (HOS) cells and magneto-mechanical actuation (MMA) for targeting and destroying HOS cells. We show that MPs are easily incorporated by ADSCs and HOS cells, as confirmed by TEM images and a ferrozine assay. MP-loaded ADSCs display increased motility towards tumor cells compared with their unloaded counterparts. MMA of MP-loaded ADSCs induces HOS destruction, as confirmed by the MTT and live/dead assays. MMA enables the release of the MPs towards cancer cells, producing a significant decrease (about 80%) in HOS viability immediately after application. In contrast, normal human dermal fibroblasts’ (NHDFs) viability exposed to similar conditions remains high, showing a differential behavior of normal and malignant cells to MP load and MMA exposure. Taken together, the method could derive successful strategies for in vivo applications in targeting and destroying malignant cells while protecting normal cells.

## 1. Introduction

The efficacy of traditional cancer treatments, including surgery, chemotherapy, and radiotherapy, along with newer methods like immunotherapy and hormone therapy, has steadily improved over the past few decades. This progress has resulted in higher survival rates and a better quality of life for oncology patients. However, there are still significant limitations to conventional therapeutic approaches, such as tumor relapses and various side effects specific to each therapeutic approach. The application of nanotechnologies, specifically magnetic micro- and nanoparticles (MPs), as versatile tools for cancer diagnosis and therapy has recently gained momentum [1,2]. Their specific magnetic properties and the possibility of manipulating them through actuation in alternative magnetic fields have led to their use in various biomedical applications [3]. One of the main approaches is hyperthermia, which increases the temperature of the cells through the actuation of the MPs dispersed in tumor tissue in the presence of an alternating magnetic field at high frequencies [4,5]. Magneto-mechanical actuation (MMA) is another application that uses MPs to destroy cancer cells by mechanically actuating MPs in low-frequency magnetic fields [6,7]. This effect relies on the spatial oscillations or rotations of MPs, which accurately follow the oscillating or rotating external magnetic fields. Consequently, a magnetic torque is generated, enabling MPs to hit the cancer cells externally or internally and leading to their death. In order to increase the magnitude of the torque acting on particles that are positioned in a magnetic field, it is necessary for the magnetic particles (MPs) to possess high saturation magnetization and high magnetic anisotropy. There are a number of research papers that detail the behavior of various types of MPs for medical applications through the magneto-mechanical effect. For instance, SPION magnetite particles and Fe-Ni-Co alloy particles that have been synthesized through chemical methods [8] as well as Fe-based particles that have been produced through high-energy ball milling [9] and disc-shaped particles that have been fabricated through the combination of vacuum-deposition techniques with lithography techniques [10,11]. Additionally, there are investigations that delve into the characteristics and properties of different kinds of magnetic nanowires (NWs) as well as magnetic nanochains and nanobundles [12,13,14].

Recently, we have found that Fe-Cr-Nb-B magnetic particles (MPs), produced by milling the rapidly solidified melt-spun ribbon precursors, have a glassy structure, cuboid shapes, and sizes between 10 nm and 200 nm and behave super-ferromagnetically [9,15]. Alloys from this family have relatively low Curie temperatures, in the range of 52–15 °C, depending on a Cr content between 11.5 and 12.5 [15]. In this study, we chose to prepare MPs with the composition Fe_68.2_Cr_11.5_Nb_0.3_B_20_, which present significant magnetic anisotropy and high saturation magnetization, characteristics that facilitate their rotation in a rotating magnetic field of relatively small intensity (less than 150 Oe) and at low frequencies (less than 5 Hz). These particles allowed us to obtain a higher reduction in the viability of cancer cells through magneto-mechanical actuation compared with other, previously tested particles [9].

To study the effect of MMA on cancer cells, we used human osteosarcoma cells (MG-63), which are a representative cell line of osteosarcoma, a form of cancer known to be highly aggressive and lead to numerous metastases [16]. Conventional therapies are not always efficient in treating this type of cancer, so we tried to develop an alternative. Since destroying the cancer cells would require the presence of therapeutic MPs at the tumor site, we studied the possibility of transporting these particles using adipose-derived mesenchymal cells (ADSCs) as carriers. These cells are known to preferentially target tumors, inflammations, or wound-healing sites, making them a convenient modality to carry therapeutic substances [17]. Magneto-mechanically actuating MP-loaded ADSCs, after insertion in the human osteosarcoma cell area, induce their death and the release of the MPs both from the surface and inside of the former cells, to be subsequently internalized by HOS. To assess the cytotoxic effect of Fe-Cr-Nb-B MPs and the magneto-mechanical actuation effect on healthy and tumor tissues, comparative experiments on HOS, ADSCs, and normal human dermal fibroblasts (NHDFs) were performed. NHDFs were used as a ubiquitous cell type present within organ stroma, musculoskeletal tissues, and tumor stroma [18].

We emphasized the effects induced by Fe-Cr-Nb-B particles in a rotating magnetic field on the viability of HOS cells and ADSCs compared with the absence of effects when testing on NHDFs. We tried to highlight to what extent the destruction of HOS cells takes place through the direct action of moving particles on the cell membrane as well as through the induction of apoptosis. In this sense, we studied the internalization of Fe-Cr-Nb-B particles inside HOS cells as well as inside ADSCs related to the transport of particles in the tumor area. Thus, ADSCs loaded with Fe-Cr-Nb-B particles were tested as a modality to transport and release particles to cultures of HOS cells. Magnetic actuation of MPs released by ADSC carriers was shown to induce HOS destruction. Here, we present in detail an optimized modality to load ADSCs with MPs, to target HOS cells using ADSC–MPs in vitro, and to actuate the magnetic particles in a rotating magnetic field. Finally, the efficiency of this approach is discussed.

## 2. Materials and Methods

The Fe_68.2_Cr_11.5_Nb_0.3_B_20_ particles were prepared through grinding. Dimensional measurements were performed using DLS and HR-SEM. Cell viability tests include MTT test, live/dead assay, LDH assay. Quantitative and qualitative presence of MPs in cells was assessed using a ferrozine assay, optical microscopy, HR-SEM, and HR-TEM. Other assays performed on cells: cell migration tests; evaluation of apoptosis markers, caspases 3/7 assay; and evaluation of the cellular cytoskeleton, phalloidin.

Magnetic particle preparation. Fe_68.2_Cr_11.5_Nb_0.3_B_20_ magnetic particles with sizes between 10 and 200 nm and cuboid forms were prepared by high-energy ball milling (Fritsch Vibratory Ball Mill, Pulverisette 7 Premium Model, Fritsch, Germany) of precursor melt-spun rapidly quenched ribbons, as described in [16]. Oleic acid was used as surfactant during the milling to avoid the particles’ surface oxidation.

Ferrofluid preparation. An amount of 80 mg of Fe-Cr-Nb-B MPs obtained by milling in oleic acid, as described above, were washed 3 times with NaOH 5% to remove the oleic acid excess from the particle surface, followed by washing with deionized water until a pH of 7 was reached [19]. MPs were separated with a magnet, and the water was removed and replaced with 1 mL of calcium gluconate solution, 94 mg/mL. The sample was ultrasonicated for 30 min at 80 °C using an ultrasonic probe. The resulting ferrofluid was sterilized at 121 °C for 30 min using an autoclave.

Vibrating-sample magnetometry (VSM). The magnetic properties of the Fe-Cr-Nb-B MPs were investigated using a 7410 Series Vibrating Sample Magnetometer (VSM) from Lake Shore Cryotronics, Inc., Westerville, OH, USA.

DLS analysis (dynamic light scattering). For DLS analysis, 5 mg of nanoparticles were dispersed in 10 mL ultrapure water under ultrasonication for 5 min. The particle concentration was about 0.5 mg/mL, and we used a sample of 2 mL. The DLS results expressed the intensity–distribution size of the nanoparticles, which showed that they are not volume- or number-related. The sample was analyzed with Microtrac/Nanotrac 252 equipment (Montgomeryville, PA, USA).

HR-SEM particle analysis. For HR-SEM analysis, the magnetic nanoparticles were diluted in ultrapure water, placed onto silicon wafers, and air-dried. The images were obtained using a Carl Zeiss NEON 40 EsB CrossBeam, Oberkochen, Germany, and its software, JEOL, for nanoparticle count and size distribution.

For the magneto-mechanical actuation of MPs, the samples were treated in 30 min intervals in a rotating magnetic field of 150 Oe at a frequency of 2 Hz.

Cell seeding and MTT assay. HOS cells, ADSCs, and NHDF cells were seeded in 96-well plates and incubated until 90% confluency. Then, the culture media were replaced in some of the wells with fresh media containing the ferrofluid. Then, 24 h after adding the particles, the MTT (3-(4,5-dimethylthiazolyl-2)-2,5-dyphenyltetrazolium bromide) assay was performed to evaluate the cellular viability. The MTT assay, which is based on the decrease of tetrazolium salts in metabolically active cells through dehydrogenase enzymes, monitored the formation of intracellular purple formazan, which could be further solubilized and quantified by spectrophotometric methods [20].

The cell viability (%) is calculated using the equation [21]:

(1) $$\mathrm{CV}\;(\%)=100\;\times \;\frac{{\mathrm{OD}}_{\mathrm{Ferrofluid}\;}-{\mathrm{OD}}_{\mathrm{Blank}}}{{\mathrm{OD}}_{\mathrm{Control}}-{\mathrm{OD}}_{\mathrm{Blank}}},$$

where CV (%) is the cellular viability and OD is the optical density of the wells containing (a) cells with ferrofluid (OD_Ferrofluid_), (b) only cells (OD_Control_), and (c) cell culture medium without cells (OD_Blank_). The sample absorbance was measured at 570 nm by means of a Multi-Mode Microplate Reader Synergy HTX, Agilent Technologies, Santa Clara, CA, USA.

HOS cells, ADSCs, and NHDF cells were seeded in 24-well plates and incubated until 90% confluency.

LDH assay (lactate dehydrogenase assay). We used 96-well plates containing ADSCs and HOS at 90% confluency, which received 1 mg/mL of Fe-Cr-Nb-B nanoparticles. After 24 h, plates underwent MMA exposure and were assessed for LDH release. LDH assay quantified the activity of the LDH cytosolic enzyme released after the rupture of the cell membrane. The released LDH was measured 12 h after MMA. Each sample consisted of five wells (*n* = 5), which included controls for both spontaneous LDH activity and maximum LDH activity. The maximum LDH activity controls were lysed with 10 µL of 10× lysis buffer, while 10 µL of water was added to each well for the spontaneous LDH activity controls. After 45 min, over 50 µL of culture medium had 50 µL of reaction mixture added from the assay kit. Then, 30 min later, 50 µL of stop solution was added, and absorbance measurements were performed at 490 nm and 680 nm. Absorbance at 680 nm was subtracted from 490 nm value. Following equation is used for cytotoxicity calculation:

(2) $$\%Cytotoxicity=\frac{Sample\;LDH\;activity-Spontaneous\;LDH\;activity}{Maximum\;LDH\;activity-Spontaneous\;LDH\;activity}\times 100$$

ADSC–MP carriers added to the HOS culture. To evaluate the efficiency of ADSCs as carriers, we plated ADSCs in 24-well plates and incubated them until they reached confluency. Afterwards, the fresh cell medium with 1 mg/mL MPs was added to each well. Following the 24 h co-incubation, the wells were trypsinized, and the cells re-suspended in a fresh medium that was added to the HOS cultured in 24-well plates until confluency. Then, 2 h after adding the ADSCs loaded with MPs onto the HOS wells, they were actuated magneto-mechanically for 30 min to destroy the ADSCs and release the MPs. The samples were left in the incubator for 24 h and further magneto-mechanically actuated to destroy the osteosarcoma cells that internalized the MPs.

Incorporation of Fe-Cr-Nb-B magnetic particles on HOS and ADSCs. Ferrozine Assay. HOS and ADSCs were plated on 24-well plates. MPs were added after 24 h, and the experiments were performed after a further 24 h to allow the cells to interact with the particles. The cells were double-washed with PBS to remove any extracellular MPs, fixed with 70% ethanol for 15 min, and again double-washed with PBS. A total of 500 μL of 50 nM NaOH was added to the wells for 2 h on a shaking plate. We used 3 wells per experiment. Aliquots of lysed cells were transferred to Eppendorf tubes of 1.5 mL and mixed with 500 μL of 10 mM HCl and 500 μL of iron-releasing reagent (a freshly mixed solution of equal volumes of 1.4 M HCl and 4.5% KMnO_4_ in distilled water). The mixture was incubated for 2 h at 60 °C in a fume hood. A total of 150 μL of iron-detection reagent (6.5 mM ferrozine, 6.5 mM neocuproine, 2.5 M ammonium acetate, and 1 M ascorbic acid, all dissolved in water) was added to each tube. After 30 min, 500 μL of the solution from each tube was transferred into a well of the 24-well plate, and the absorbance measured at 570 nm with a spectrophotometer. For the calibration curve, we used FeCl_3_ standards (0 ÷ 300 µM) in 10 mM HCl to allow the calculation of iron content per sample.

Live/dead assay. The cells were seeded in a 24-well plate until reaching 90% confluency. The MPs were added afterwards, and the plate was left for another 24 h in the incubator. For HOS–MPs and ADSC–MPs, we proceeded with the assay, while for the HOS–ADSC–MPs, we trypsinized ADSC–MPs, added them onto the HOS wells, and left them for 2 h in the incubator. Following this time interval, they were actuated magneto-mechanically for 30 min to destroy the ADSCs and release the MPs. The samples were left in the incubator for 24 h and further actuated magneto-mechanically to destroy the osteosarcoma cells that internalized the MPs. For this assay, the cells were washed with PBS, incubated for 30 min at room temperature with diluted live/dead assay reagents, and visualized under EVOS inverted light microscope, AMG (Advanced Microscopy Group), USA, under GFP and RFP fluorescence filters.

The FAM-FLICA caspase assay. This kit consists of a fluorescent dye that binds nucleic acid. In apoptotic cells with activated caspase-3/7, the DEVD peptide is cleaved, which allows the dye to bind to DNA and emit bright green fluorescence. The reagent was diluted in PBS with fetal bovine serum dilution 1:30 *v*/*v*. Cell culture medium was removed, and the cells were incubated with 100 μL of diluted reagent at 37 °C and 5% CO_2_ for 30 min. To evaluate the results, the plates were read in the plate reader at excitation 485 and emission 520.

In vitro cell motility. Part of the HOS cell sheet from the Petri dishes was removed using a custom-made scrapper in order to create a cell-free zone of about half the area of the dish, double-washed with PBS and complete cell media. ADSCs and ADSCs loaded with MPs were added to HOS in the Petri dishes. After 20 ÷ 24 h, a “gap” of about 1 mm was created between the two cell populations using a scraper. The cells were placed in a custom-made mini-incubator and observed with a fluorescent inverted microscope for 24 h. The images were taken at intervals of 10 min, and processed using image software (ImageJ 1.52a).

Electron microscopy for MP-loaded HOS and ADSC samples. Scanning electron microscopy (SEM) was performed using an adaptation of the protocol described by Fischer et al. [22]. HOS and ADSCs were grown on sterilized silicon chips placed in Petri dishes until 80% confluence and incubated with MPs for 24 h. Afterwards, the chips’ surfaces were washed with PBS to remove the non-attached MPs, and the cells were fixed with glutaraldehyde solution and osmium tetroxide solution. Following these steps, the cells were dehydrated using ethanol solutions in increasing concentrations, followed by air drying in the biological safety hood and in vacuum. Then, the silicon chips were coated with a 5 nm film of Au and investigated using a Carl Zeiss NEON 40 EsB CrossBeam system, GmbH, Oberkochen, Germany.

The preparation of samples for transmission electron microscopy (TEM) was performed using the protocol described by Schrand et al. [23]. For this, HOS and ADSCs were grown in 25 mL Corning flasks to 80% confluence and incubated with 200 µg/mL MPs for 24 h. After the incubation, the supernatant was removed and the cells washed 2 times with PBS to remove the excess particles, then trypsinized and centrifuged to form a pellet on the bottom of a Corning 15 mL centrifuge tube. The pellet was fixed with a mixture of fresh 2.5% glutaraldehyde/paraformaldehyde solutions for 2 h at room temperature. After the fixation was complete, the sample was rinsed with PBS and treated with 1% osmium tetroxide in PBS for another 1 h. Then, the fixative was removed by repeated washing of the probe with PBS and water. The probe was later dehydrated using a graded series of ethanol concentrations (50, 70, 90, and 99%), followed by embedding the sample in epoxy resin, cutting it with an ultra-microtome, and visualizing it using a UHR-TEM model LIBRA^®^ 200 MC from Carl Zeiss GmbH (Jena, Germany).

Assessment of HOS and ADSC cytoskeleton fibers. The cells were plated in 35 mm Petri dishes in complete cell media. After 24 h, 1 mg/mL MPs were added to the complete cell culture media and co-incubated for a further 24 h. HOS and ADSCs with or without MPs were exposed to MMA for 30 min. The cells were fixed with 2% paraformaldehyde for 15 min, stained with phalloidin (Texas Red™-X Phalloidin Termo Fischer Scientific), counterstained with DAPI, and visualized using an EVOS inverted light microscope under RFP fluorescence filter.

Statistics. All experiments were performed in biological triplicate. One way analysis of variance (ANOVA) and Bonferroni post hoc analysis were performed using OriginLab PRO version 8.0; between-group and within-group differences were considered significant at *p* ≤ 0.05 (*n* = 5).

## 3. Results and Discussion

### 3.1. Preparation of Fe-Cr-Nb-B Magnetic Particles

Fe_68.2_Cr_11.5_Nb_0.3_B_20_ MPs were obtained by high-energy ball milling from melt-spun amorphous ribbon precursors. The preparation of Fe-Cr-Nb-B magnetic particles involves making an ingot with the desired composition by vacuum melting of the component elements, followed by obtaining ribbons by rapid quenching of the melt on a rotating copper disc to ensure a high cooling speed that allows the solidification of the ribbons in an amorphous state. The amorphous state gives the ribbons specific magnetic properties, such as high saturation magnetization obtained at low magnetic fields and low Curie temperatures dependent on the Cr content in the alloy. The amorphous ribbons are heat-treated for embrittlement, then subjected to a mechanical grinding process in high-energy mills in a humid environment until nanometer-sized particles are obtained, particles that preserve the initial amorphous state. The concentrations of Fe, Cr, and Nb in the nanoparticles were measured with an energy-dispersive X-ray spectroscopy (EDX) system attached to the Carl Zeiss Libra 200 MC Transmission Electron Microscope. The amount of B was quantified using the Perkin-Elmer AANALYST 200 Atomic Absorption Spectrometer (AAS). The details about their preparation are given elsewhere [9,15]. They have non-zero coercivity at room temperature, large magnetic susceptibility, and a low Curie temperature [15]. The Fe-Cr-Nb-B MPs were used to prepare a ferrofluid (FF), which ensures good dispersion and prevents agglomeration [19] when the MPs are added to the cell culture medium. Calcium gluconate was used as a dispersion medium due to its biocompatibility [24].

**Figure 1:**
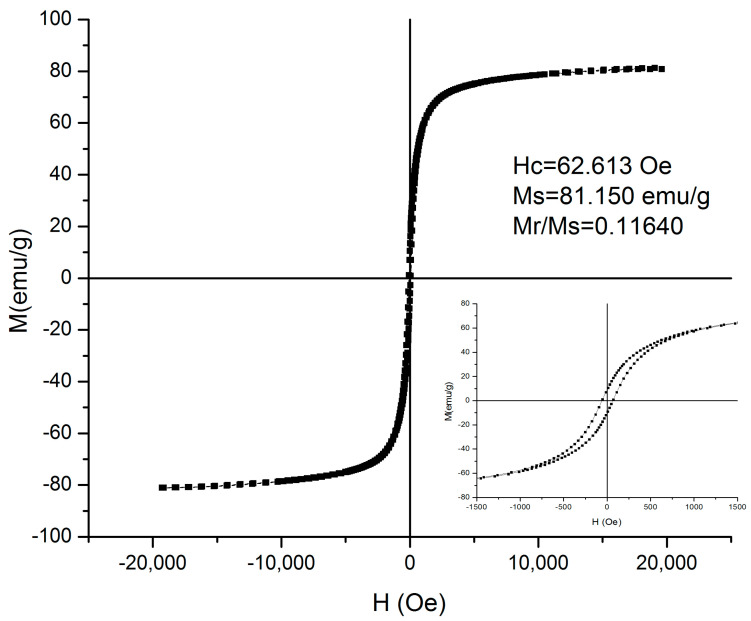
Magnetic hysteresis loop for Fe_68.2_Cr_11.5_Nb_0.3_B_20_ MPs. The measurement was performed at room temperature.

Figure 1 presents the hysteresis cycle for particles with the composition Fe_68.2_Cr_11.5_Nb_0.3_B_20_. The magnetization of saturation has a value of 81.15 emu/g and a reduced value of magnetic remanence. Reaching saturation at relatively low values of the applied magnetic field indicates the high magnetic susceptibility of these particles. Measurements of the temperature dependence of the saturation magnetization show that the alloys used in these experiments have a Curie temperature of 52 °C, which is typical for the Fe-Cr-Nb-B system, which is known for its low Curie temperatures.

**Figure 2:**
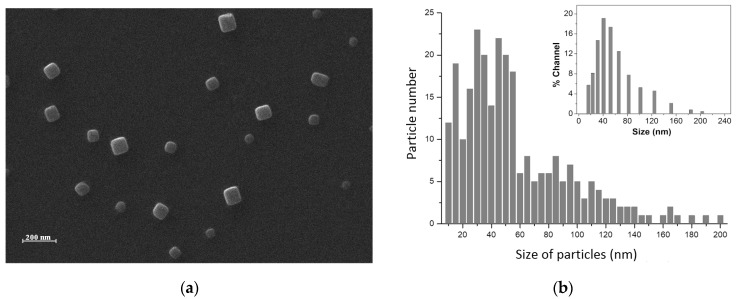
(**a**) HR-SEM image for Fe_68.2_Cr_11.5_Nb_0.3_B_20_ particles; (**b**) Size distribution of Fe-Cr-Nb-B MPs. Inset: DLS histogram of the nanoparticles. The particle concentration was about 0.5 mg/mL.

The scanning electron microscopy (SEM) images recorded for the obtained magnetic particles show a defined, parallelepiped shape. The magnetic particles in the sample reveal a pattern with a very uniform morphology, ranging in size from 30 to 200 nm. The size of most of the particles was below 100 nm in diameter, as measured by SEM and dynamic light scattering (DLS) (Figure 2a). The statistical distribution of the particle size was determined using JEOL software that analyzes SEM images. More results are shown in the figure below.

As Figure 2b shows, there is a relatively good match between the size distributions of the nanoparticles. However, the DLS analysis resulted in a better Gaussian distribution of the dimensions of the nanoparticles, most probably due to the increased number of particles analyzed through DLS, which highly improved the statistical calculus and reduced the errors. The polydispersity index (PDI) obtained from DLS data was lower than 0.1, indicating a narrow size distribution.

### 3.2. Biocompatibility of Fe-Cr-Nb-B Magnetic Particles

The potential use of magnetic particles for the destruction of cancer cells by magneto-mechanical effect implies that these MPs are biocompatible and do not elicit cytotoxicity for different cell types.

**Figure 3:**
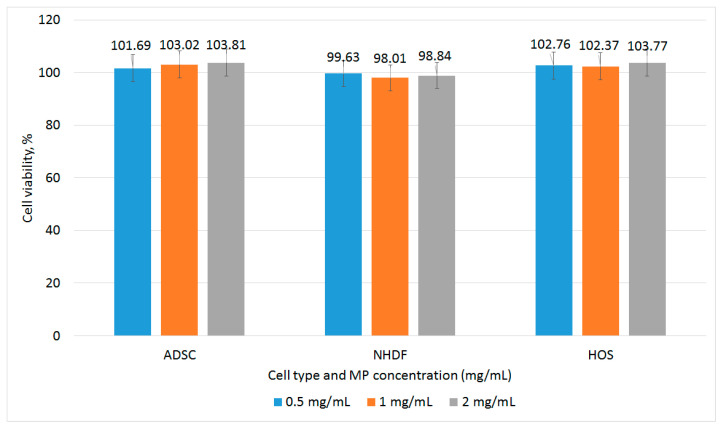
Relative cell viability of ADSC, NHDF cell, and HOS cell cultures after 24 h co-incubation with various concentrations of Fe-Cr-Nb-B MPs assessed with MTT assay. The nanoparticles show good biocompatibility. No statistically significant differences were observed at *p* ≤ 0.05.

Prior to the exposure to variable magnetic fields, the ferrofluid containing Fe_68.2_Cr_11.5_Nb_0.3_B_20_ MPs was evaluated indirectly by measuring the proliferation rate of the cells exposed to increasing concentrations (between 0.5 and 2 mg/mL) of MPs added in the cell culture media. The tests were performed on several different cell types: a tumor cell line-human osteosarcoma (HOS) cell, and two normal primary cell types: primary human adipose-derived mesenchymal cells (ADSCs) and primary normal human dermal fibroblasts (NHDF). The cell viability was assessed using the MTT (5-dimethylthiazol-2-yl-2, 5-diphenyltetrazolium bromide, Vibrant ^®^TermoFisher Scientific, Waltham, MA, USA) assay. No cytotoxic effect was observed, even for MP concentrations as high as 2 mg/mL (Figure 3).

These particles were previously evaluated for biocompatibility, and they showed no cytotoxic effects on any of the cells that they were tested on, even when higher concentrations were used, such as 5 mg/mL [9,19].

### 3.3. The Effect of Magneto-Mechanical Actuation on HOS, ADSCs, and NHDF

Excellent cell viability results provided information that MPs alone do not induce cytotoxicity and can be employed for further tests.

The experimental setup for magnetic field actuation consists of a custom-made system of four (4) coils placed in a cross, which can produce a uniform rotating magnetic field between 10 Oe and 200 Oe in a given volume [9]. The free space inside the four-coil system is about 20 cm^3^, allowing the placement of the cell culture plates. The system allows the setup of the magnetic field intensity and frequency, as well as the time of exposure. The temperature control device permits the modification and maintenance of the desired temperature of the cell plates. The coil system is powered by two waveforms phase-shifted by 90 degrees. In this way, they fit with the orthogonal arrangement of the coil system, allowing the resultant magnetic field to rotate with the frequency of the waveforms. The frequency and amplitude of the waveforms can be adjusted in a wide range, from a few mHz up to several kHz. Culture cell plates were placed inside the coil system in a uniform magnetic field area and exposed to the rotating magnetic field, determining MP movement and subsequently cell destruction and death. The evaluation of cell viability after the experiment was made by using an MTT test.

**Figure 4:**
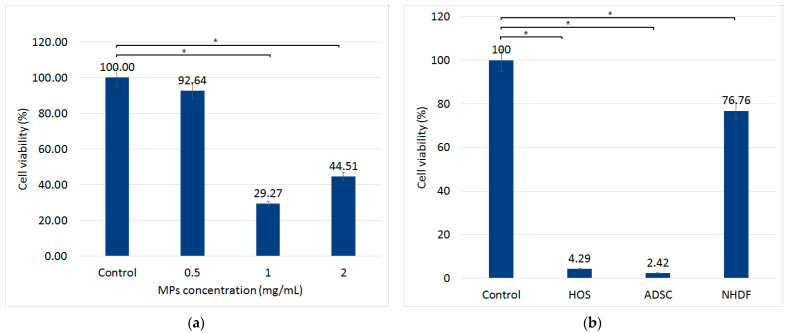
(**a**) HOS viability following magneto-mechanical actuation (MMA) for different concentrations of MPs. A significant decrease in cell viability is observed after a 30 min session of MMA with 1 mg/mL MPs compared with control and 0.5 mg/mL MP concentration samples. (**b**) Cell viability following double MMA session of HOS, ADSCs, and NHDF co-incubated with 1 mg/mL of MPs. MTT assay was performed after a 24 h time period. * = significance bars at *p* ≤ 0.05.

Figure 4a shows the variation of the viability in HOS following magneto-mechanical actuation for 30 min. Cells were previously incubated with MPs for 24 h. Cell culture media with MP concentrations ranging from 0.5 to 2 mg/mL were added to the plate wells. There was a significant decrease in cell viability, which reached the minimum for MP suspension with a concentration of 1 mg/mL. It is noteworthy that, when exposed to MMA, a higher MP concentration (2 mg/mL) induces a significant decrease in HOS viability; however, it is less than that caused by 1 mg/mL. This effect can be explained by the increased density of MPs per volume unit, which impedes the free rotational movement that would induce the cells’ destruction. Given these results, all subsequent experiments were performed using a concentration of 1 mg/mL MPs added to cell culture media.

After exploring which concentration of MPs delivers the highest level of destruction on HOS under MMA, we evaluated the effects of the treatment on HOS, ADSCs, and NHDF (Figure 4b) using the same concentration of MPs but with two magneto-mechanical actuations performed 24 h after co-incubation, with 24 h between each treatment. For HOS and ADSCs, doubling the number of MMA treatments leads to viability under 5%, while for NHDF, the viability is above 75%, showing that normal cells are not as affected by these treatments.

### 3.4. Internalization of Fe-Cr-Nb-B Magnetic Particles by HOS and ADSCs

We further investigated the possibility of transporting the Fe-Cr-Nb-B MPs using ADSCs in the area of cancer cells and the effect of magneto-mechanical actuation on inducing particle release from ADSC carriers for their direct action on cancer cells. We tested the feasibility of incorporating Fe-Cr-Nb-B MPs within ADSCs as carriers and studied MPs’ cell load by SEM and transmission electron microscopy (TEM), as well as by assessing cellular iron content using a ferrozine assay. Similar tests were performed to evaluate the presence of MPs inside HOS in order to determine how magneto-mechanical actuation interferes with cell viability.

**Figure 5:**
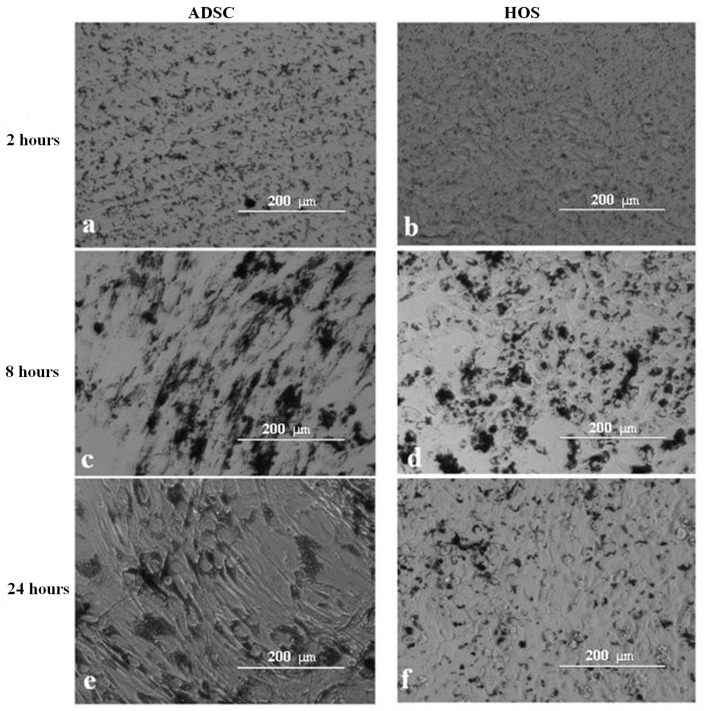
ADSC (**a**,**c**,**e**) and HOS (**b**,**d**,**f**) at different intervals (2, 8, and 24 h) following MP addition within the cell culture medium. Images were taken with an inverted light microscope in bright field, magnification 20×.

Following addition to the cell culture medium, MPs were routinely evaluated using an inverted optical microscope. We examined the interaction between MPs and cells at different time intervals after their addition. As it can be seen in Figure 5a,b, 2 h after their addition, MPs are dispersed within the cell culture medium. After 8 h, MPs start to sediment on the cells’ membrane, and the process of MP incorporation inside the cells begins (Figure 5c,d). The MPs are completely attached to the cell membranes 24 h after their addition (Figure 5e,f). Following these observations, we decided to perform MMA 24 h after MP co-incubation.

**Figure 6:**
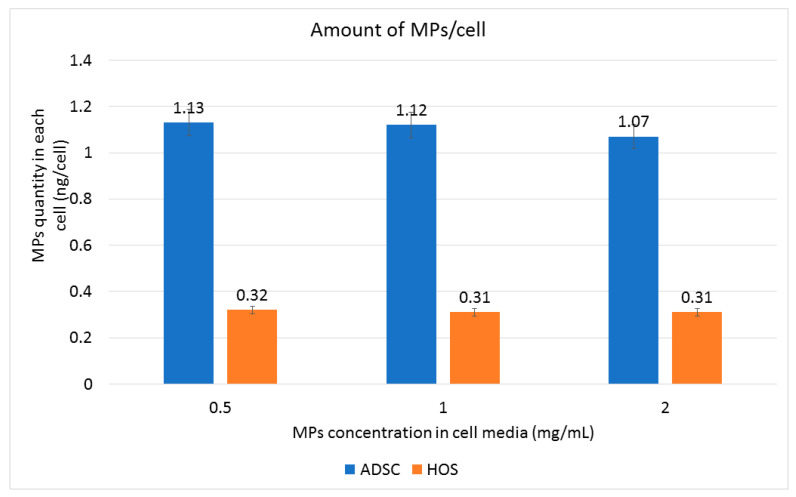
Ferrozine assay quantifying ADSC and HOS intracellular MP content after 24 h of co-incubation with Fe-Cr-Nb-B magnetic particles.

**Figure 7:**
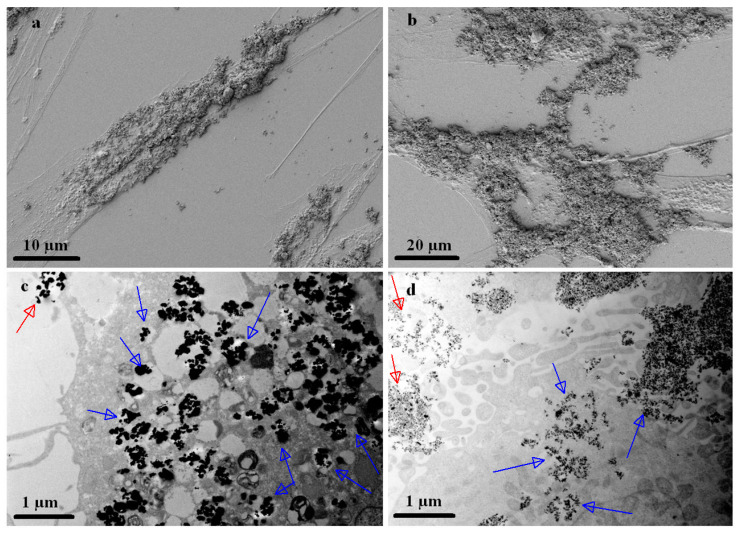
HR-SEM images of ADSCs (**a**) and HOS cells (**b**) covered with MPs. HR-TEM images of ADSCs (**c**) and HOS cells (**d**), in which internalized MPs can be observed. The magnetic nanoparticles are localized outside (on the cell membrane: (**a**,**b**)) and also inside the cell, forming nanoparticle vesicles (**c**,**d**). Red arrows point to MPs that are in the exterior of the cells, and blue arrows point to internalized MPs.

The ferrozine assay was performed in order to evaluate the presence of MPs inside the cells. The quantity of iron per cell was determined 24 h after adding the MPs to the culture medium. We calculated the iron content in cells loaded with MPs after subtracting the amount of physiological iron cell content in non-loaded ADSCs and HOS cells in similar culture conditions. ADSCs, being dimensionally larger, were found to incorporate higher amounts of MPs (1.12 ng of iron per cell) than HOS cells (0.31 ng of iron per cell) (Figure 6), but both types of cells appear to be saturated with particles, both inside (Figure 7c,d) and outside, on the cell membrane (Figure 7a,b).

It is noteworthy to mention that the quantity of MPs added to the cell media has little effect on the quantity of MPs found in a cell. MP internalization, which is an active membrane process, is performed as a function of time. Indeed, the duration of interactions between cells and particles rather than their concentration in the culture medium has been found to influence particle upload [25]. The quantity of MPs that the cells internalized appears to be enough for the process of MMA to affect the loaded cells.

To evaluate deeper the interaction between cells and MPs and demonstrate the MPs’ internalization, we have used scanning (SEM) and transmission (TEM) electron microscopy. MPs appear to adhere to the surface of the cell membrane when co-incubated for 24 h, despite the numerous washes required by sample preparation for SEM investigation (Figure 7a,b). MPs appear to be incorporated within ADSCs as well as HOS cells (Figure 7c,d). Since ADSCs are larger cells, they can incorporate higher quantities of MPs compared with HOS cells. However, both cell types were demonstrated to internalize the MPs. MPs can be observed within the lysosomes in both cell types, being probably further trafficked inside the cells’ cytoplasm without trespassing into the cellular organelle membrane.

Taking these results into account, we can conclude that MPs not only adhere to the cell surface but are also internalized by the cells without damaging the cell membrane. These findings offer a solid foundation for demonstrating that magneto-mechanical actuation of MPs while in contact with the cell membranes or internalized by the cells strongly affects the cells’ viability by means of various membrane and possible lysosomal disruption mechanisms.

### 3.5. ADSC Motility for Targeting HOS Cells In Vitro

Cell motility was studied in order to see if and how ADSCs, both loaded and unloaded, move towards a cell front, particularly towards HOS.

**Figure 8:**
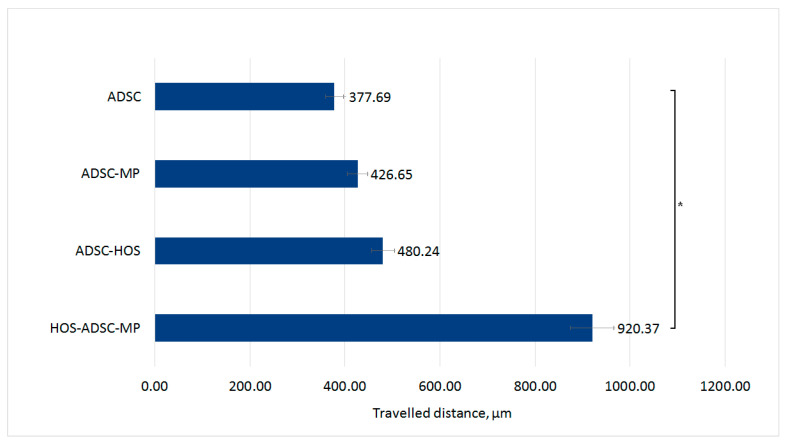
Cell motility of MP-loaded and un-loaded ADSCs in a “wound-healing model’’. Traveled distance in µm recorded over 24 h live imaging process and data processed with Image J 1.52a software. * = significance bars for *p* ≤ 0.05.

The cell motility was evaluated in vitro using a modified “wound healing assay”, while the live cell imaging was studied by using an inverted microscope (live imaging). The migration of ADSCs loaded with MPs towards the tumor cell zone was recorded using a time-lapse cell imaging method and computational interpretation of the results (Image J). Before evaluation, the two cell populations (ADSC–MPs and HOS) were separated by a free space of about 0.5 mm by using a pipette tip (“wound healing” model). The image analysis revealed that MP-loaded ADSCs were able to target the osteosarcoma cells. Moreover, the calculated cell speed of ADSC–MPs was almost double compared to unloaded ADSCs (control sample) in similar culture conditions (Figure 8).

We found that unloaded ADSCs have the lowest speed in the given experimental conditions. The explanation might reside in the fact that random movements are specific for adherent cells in two-dimensional cultures, and the cells tend to occupy the empty spaces within the culture dish and to mechanically “close” the gap. The unloaded ADSCs move the shortest distance in the 24 h interval: ~380 µm. In the case of ADSCs with MPs, they move a slightly higher distance, covering about 430 µm in 24 h. This difference might be explained by the possible increased mitochondrial metabolism due to the iron intake, leading to higher viability as well as increased motility. It is possible as well that Fe-Cr-Nb-B MPs indirectly promote cell movement by means of an increased cell proliferation rate. When ADSCs were co-cultured with HOS, the distance covered further increased to about 480 µm in 24 h. Recently, it has been reported that tumor cells’ specific cytokine release (such as tumor necrosis factor (TNF)-α and the interleukin family) activates the migratory mechanisms in mesenchymal stromal cells by increasing the homing responsive surface receptors such as CXCR4 or CXCR7 [26]. In this case, the co-culture environment could have increased the ADSC speed, most probably by providing the necessary soluble stimuli inside the culture medium. Moreover, the cells were found to orient themselves towards “the tumor” (in this case, the HOS cells’ front), showing a targeting potential dependent on the HOS-released cytokine gradient.

The highest recorded cell speed was in MP-loaded ADSCs in co-culture with HOS, which was significantly higher compared to non-loaded ADSCs. The distance covered in this case was about 920 µm in 24 h, almost double compared with non-loaded ADSCs in co-culture with HOS. We have previously reported that several types of mesenchymal stromal cells loaded with Fe-Cr-Nb-B MPs preserve or even improve the most relevant “stemness” characteristics (such as proliferation and differentiation). Recent reports pointed out that image-based identification of cultured stem cells is correlated with their stemness and proliferative capabilities [27]. The superior cell motility displayed by MP-loaded ADSCs compared with unloaded ones could be generated by an overall increase in their characteristic phenotypic features produced by particle internalization. Evidence exists as well about an increased expression of chemokine receptors responsive to their homing and targeting capabilities in mesenchymal stem cells loaded with magnetic particles [28]. Modified surface receptor expression in Fe-Cr-Nb-B-loaded ADSCs needs to be further investigated. However, our results clearly support the superior mobility and targeting potential of MP-loaded cells.

### 3.6. Effect of Magneto-Mechanical Actuation of Fe-Cr-Nb-B Magnetic Particles Transported by ADSCs on the Viability of HOS

The aim of our study was to efficiently decrease the cancer cells’ viability by actuating Fe-Cr-Nb-B MPs in a rotating magnetic field and to design an adaptable method for transporting the magnetic particles. For this purpose, we tested the effect of magneto-mechanical actuation of Fe-Cr-Nb-B particles transported by ADSCs on human osteosarcoma cells in an in vitro model of cell interaction by loading ADSCs with MPs and delivering them to HOS cells, followed by the magnetic actuation of the cell mixture. We used the protocol of double MMA delivery, consisting of two-stage actuation: the first actuation took place a few hours after cell–MP co-incubation, while the second actuation was performed 24 h after the first.

**Figure 9:**
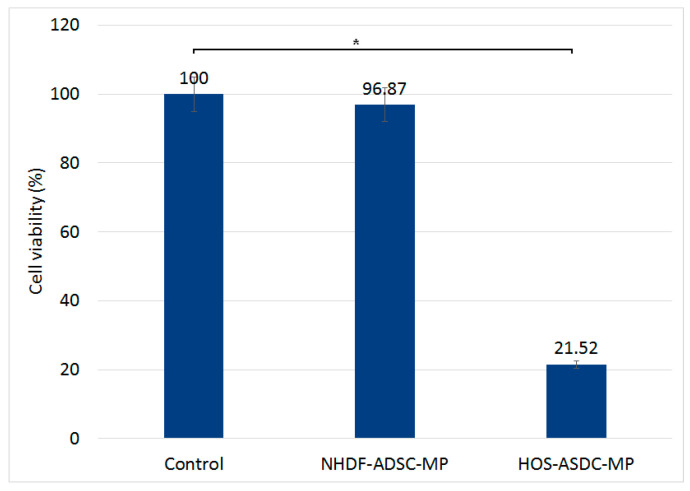
HOS and NHDF viability after MMA of cell mixture: unloaded HOS/NHDF and MP-loaded ADSCs. Viability results obtained by MTT assay 24 h after MMA exposure. * = significance bars at *p* ≤ 0.05.

Using this protocol, MMA was first performed on ADSC–MPs to evaluate only if the MPs could be released by actuation. Figure 4b shows that MMA decreases the ADSC–MP viability to ~2%, which confirms that MPs are released almost completely after the actuation. For the main experiment, MMA was applied to the ADSC–MP–HOS cell assembly under similar conditions. The loaded ADSCs (ADSC–MP) are added to the HOS cell culture and incubated for 1 h. After the incubation period, the samples with HOS and ADSC–MP are magneto-mechanically actuated for 30 min to destroy the ADSCs, which in turn releases the MPs. The samples are further co-incubated for 24 h for the internalization of MPs by HOS, after which they are magneto-mechanically actuated two more times to destroy the HOS cell culture. The results shown in Figure 9 highlight that the cell viability of HOS–ADSC–MP decreases to 21% after actuation. To ensure that MMA actuation, if used as a therapy, will not affect the normal tissue around the cancer area, a similar experiment was performed, replacing HOS cells with NHDF cells. In this case, the cell viability after double MMA exposure dropped by only 3%, a small proportion of cells being affected compared with the HOS ones.

Magneto-mechanical actuation led to the destruction of ADSC–MPs in suspension and to the release of MPs on adherent HOS cells. Given the time in culture, we can approximate that HOS cells were able to incorporate the released MPs (Figure 5). MMA delivery resulted in HOS viability dropping down to 21–22%. The irregular shape of the MPs combined with their rotational motion in the rotating magnetic field produced irreversible damage to the cell membranes and organelles and, subsequently, the cells’ death.

**Figure 10:**
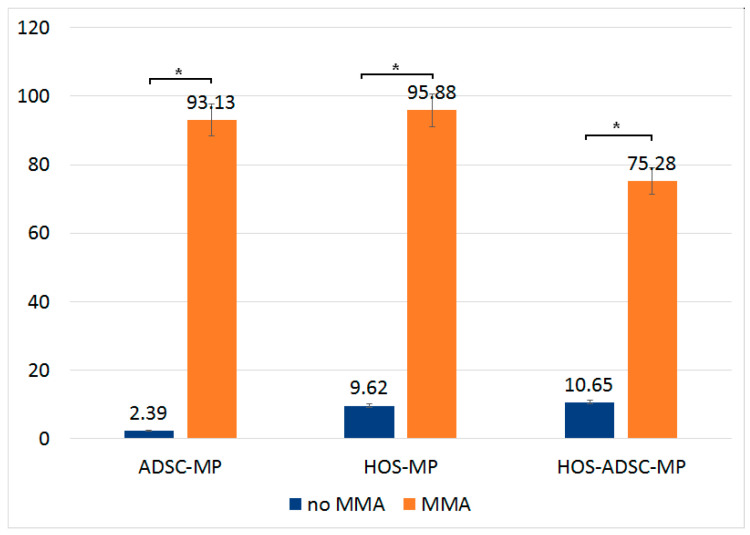
LDH assay of ADSCs with MPs, HOS with MPs, and HOS–ADSC–MPs, following MMA. After applying magnetic field, significantly higher cytosol leakage was identified, suggesting cell membrane rupture. * = significance bars at *p* ≤ 0.05.

An LDH assay (lactate dehydrogenase assay) was performed on 1 mg/mL Fe-Cr-NB-B-loaded ADSCs and HOS and also on transported ADSC–MP onto HOS culture 24 h after MMA application. Figure 10 shows the number of cells (%) presenting cell membrane rupture and cytosol leakage, subsequently confirming the MTT data previously presented. The LDH assay is usually seen as a mirror of the MTT assay as it illustrates how many cells are non-viable in the evaluated samples as opposed to MTT, which shows how many are viable.

**Figure 11:**
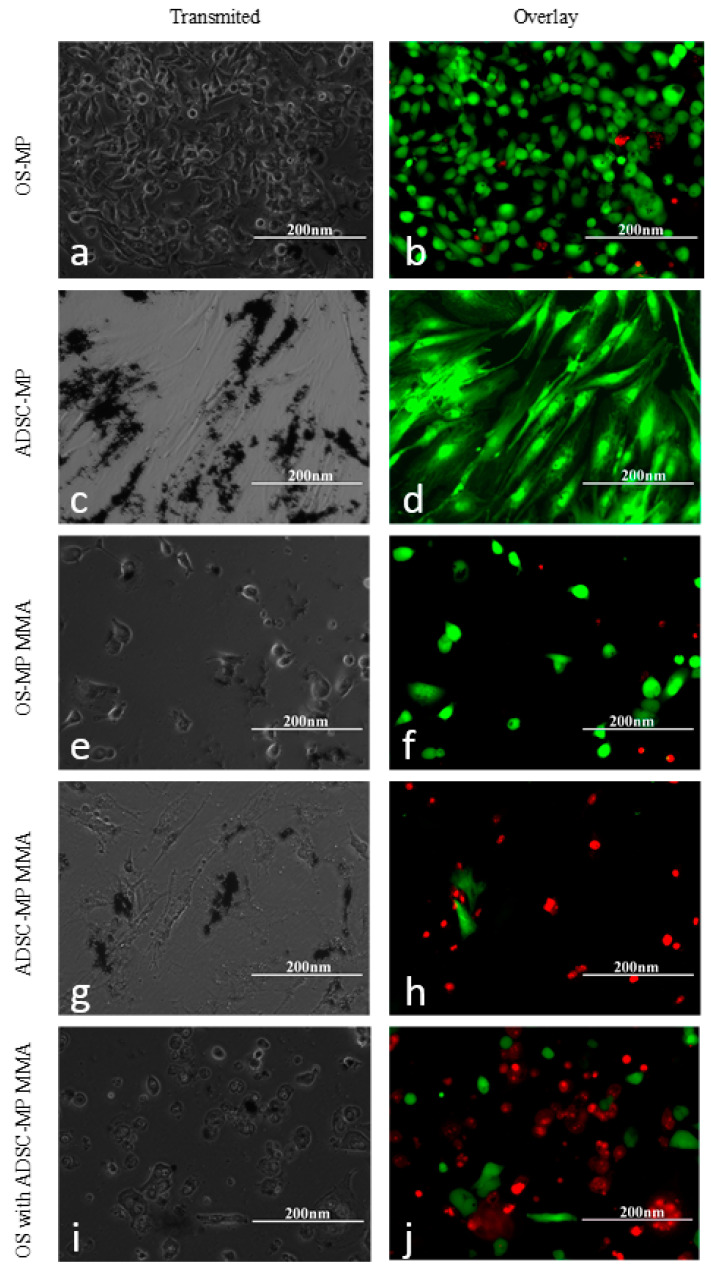
Live–dead assay of ADSCs with MPs, HOS with MPs, and HOS–ADSC–MPs, following MMA. Images taken with inverted microscope in bright field and in overlay (composed of GFP fluorescence filter for live cells (green) and RFP fluorescence filter for dead cell nuclei (red)). (**a**,**b**) HOS with MPs; (**c**,**d**) ADSCs with MPs; (**e**,**f**) HOS with MPs following MMA; (**g**,**h**) ADSCs with MPs following MMA; (**i**,**j**) HOS with ADSC–MPs following MMA.

We performed the live/dead assay [29] before and after exposure to magneto-mechanical actuation to further confirm the results presented above. The overlaid images of live (green cell cytoplasm) and dead (red cell nucleus) cells give information about the overall cell viability. ADSCs and HOS loaded with MPs display very good viability, as demonstrated by their green appearance, and the particles’ presence does not interfere with the cell viability for either type of cell (Figure 11). However, after MMA exposure, only a few cells colored green can be observed compared with the sample that was not subjected to these treatments, with most being colored red (dead cells), demonstrating reduced cell viability after the procedure, most probably caused by the interference with the cells’ membrane. Furthermore, the area without cells is bare because some of the dead cells are washed away in the process of fluorescent staining. These results further confirm that magneto-mechanical actuation of Fe-Cr-Nb-B magnetic particles carried by ADSCs strongly affects the viability of HOS, making it a viable tool for cancer treatment in the future.

These results support the feasibility of using ADSCs as carriers for Fe-Cr-Nb-B MPs. The carrier cells, which exhibit a good tumor-targeting capability, have the potential to target the tumor sites in vivo. Such cells can be safely used, as they can be easily destroyed using magnetic actuation.

**Figure 12:**
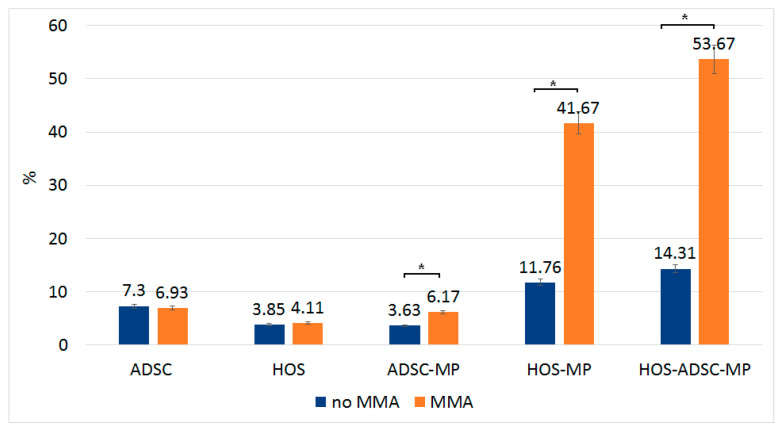
ADSC/HOS control, MP-loaded ADSC/HOS, and transported MP by ADSCs onto HOS culture representing caspase 3/7 markers after apoptosis assay. Percentage (%) of cells with caspase 3/7 fluorescence; * = significance bars at *p* ≤ 0.05.

We also evaluated the presence of apoptotic markers in the cells used for these experiments (Figure 12). For this purpose, we performed an assay to quantify the number of cells with caspase 3/7. In this case, we highlighted that the highest percentage of caspase is found in HOS cells to which ADSCs with MPs were added and that were magneto-mechanically actuated. Another high percentage was found in HOS cells with MPs after MMA. For the rest of the cell types, the percentage of apoptotic cells does not exceed 11% (considering that normal healthy cells also present a small quantity of caspase due to the ending of the cell life cycle), including ADSC–MPs cells, even when MMA was performed.

MPs are internalized by the cells through the active membrane mechanism that results in their incorporation within acidic lysosomes, as also outlined by HR-TEM [30]. MPs inside a cell body gain a rotational motion under MMA. Given that MPs are located near the cell membrane, with some also in the lysosomal compartment, MPs might disrupt the cell/lysosomal membrane, followed by leakage and triggering cell apoptosis. When MPs within these vacuoles are actuated, they can damage the membrane, leading to the release of the lysosomal content inside the cytoplasm and, consequently, to the irreversible cell damage that initiates the apoptotic cascade explained by the caspase tests [31].

**Figure 13:**
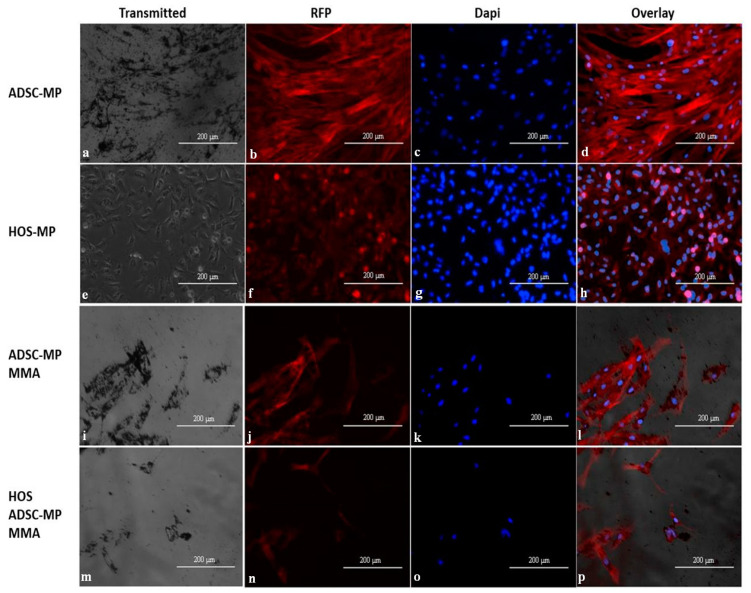
Phalloidin staining of cytoskeleton fibers of ADSCs and HOS with Texas Red™-X Phalloidin stain before and after MMA exposure. Images taken with inverted light microscope in bright light filter, RFP fluorescence filter (red cytoskeleton fibers), DAPI filter (blue cell nuclei) and overlay. (**a**–**d**) ADSCs with MPs; (**e**–**h**) HOS with MPs; (**i**–**l**) ADSCs with MPs following MMA; (**m**–**p**) HOS with ADSC–MPs following MMA.

The cells’ cytoskeleton was qualitatively investigated, as it is an important component that can be damaged by the magneto-mechanical actuation of internalized MPs. ADSCs were stained with cytoskeletal-specific phalloidin and visualized using a fluorescent microscope (Figure 13). For ADSC–MPs, the actin fibers were found to be elongated and continuous, while for HOS–MPs, the actin fibers were shorter. Following MMA, MP-loaded ADSCs appear to have shorter and uneven actin fibers. In the case of HOS exposed to MMA, the lack of continuity of the actin fibers, in addition to the cells’ disappearance, confirms that most probably the cells have been washed away following cell death.

Thus, we have demonstrated that MP cargo can be magnetically actuated in place, acting to destroy the cancer cells and may contribute to the tumor’s clearance. ADSCs can be successfully used as carriers to carry MPs to tumor sites by means of local, regional, or even systemic administration, as the cells have good tumor-targeting capabilities and can be easily destroyed by MMA after reaching the tumor site.

## 4. Conclusions

Through this research, we highlighted an in vitro cancer cell destruction method using magneto-mechanically actuated Fe-Cr-Nb-B magnetic particles. These MPs displayed good biocompatibility and a non-cytotoxic effect on both cell cultures, ADSCs and HOS. The ferrozine assay, HR-SEM, and HR-TEM confirmed the internalization of magnetic nanoparticles in vesicle form, as well as their attachment to the cell membrane. Due to the increased size and volume of ADSCs, the quantity of MPs carried by them is significantly higher compared to HOS cells. ADSCs can be safely used as carriers of Fe-Cr-Nb-B magnetic particles to cancer cell sites, a fact that could be used to design simple and convenient targeted antitumor therapies. MPs are able to induce cancer cell death after MMA, targeting several cellular components, such as the cell membrane, lysosomal compartment, and cytoskeleton. We have demonstrated that normal fibroblast cells exposed to similar experimental conditions do not significantly decrease viability compared to HOS, a fact that supports the selective action of MMA on the tumoral compartment and not on normal tissues. While these findings need to be validated in vivo, the present results are encouraging in demonstrating a modality of targeted transport of MPs by local, regional, or even systemic delivery of ADSCs loaded with MPs. The innate ability of ADSCs could be used to target tumors that are difficult to access or multiple tumoral sites.

## Data Availability

The data presented in this study are available on request from the corresponding author.
